# The impact of *Spodoptera exigua* herbivory on *Meloidogyne incognita*-induced root responses depends on the nematodes’ life cycle stages

**DOI:** 10.1093/aobpla/plaa029

**Published:** 2020-06-24

**Authors:** Crispus M Mbaluto, Esraa M Ahmad, Melody Fu, Ainhoa Martínez-Medina, Nicole M van Dam

**Affiliations:** 1 Molecular Interaction Ecology, German Centre for Integrative Biodiversity Research (iDiv) Halle-Jena-Leipzig, Leipzig, Germany; 2 Institute of Biodiversity, Friedrich-Schiller-Universität-Jena, Jena, Germany; 3 Department of Genetics, Faculty of Agriculture, Cairo University, Giza, Egypt; 4 Faculty of Land and Food Systems, University of British Columbia, BC, Canada; 5 Plant-Microorganism Interaction Unit, Institute of Natural Resources and Agrobiology of Salamanca (IRNASA-CSIC), Salamanca, Spain

**Keywords:** Above-ground–below-ground interaction, phytohormones, root-knot nematodes, *Solanum lycopersicum*, *Spodoptera exigua*, steroidal glycoalkaloids, systemic-induced responses

## Abstract

Induced responses to above-ground and below-ground herbivores may interact via systemic signalling in plants. We investigated whether the impact of above-ground herbivory on root-knot nematode-induced responses depends on the nematode’s life cycle stages. Tomato plants were infected with the nematode (*Meloidogyne incognita*) for 5, 15 or 30 days before receiving *Spodoptera exigua* caterpillars above-ground. We collected root materials after 24 h of caterpillar feeding. We investigated phytohormones and α-tomatine levels, and the expression of defence and glycoalkaloid metabolism (GAME) marker genes in tomato roots. Nematode infection alone increased the endogenous root levels of jasmonic acid (JA), salicylic acid (SA), abscisic acid (ABA), α-tomatine and the expression of the *GLYCOALKALOID METABOLISM 1 (GAME1)* gene mostly at 30 days post-nematode inoculation. Caterpillar feeding alone upregulated *Lipoxygenase D* and downregulated *Basic-β-1-glucanase* and *GAME1* expression in roots. On nematode-infected plants, caterpillar feeding decreased JA levels, but it increased the expression of *Leucine aminopeptidase A*. The induction patterns of ABA and SA suggest that caterpillars cause cross-talk between the JA-signalling pathway and the SA and ABA pathways. Our results show that caterpillar feeding attenuated the induction of the JA pathway triggered by nematodes, mostly in the nematodes’ reproduction stage. These results generate a better understanding of the molecular and chemical mechanisms underlying frequent nematode–plant–caterpillar interactions in natural and agricultural ecosystems.

## Teaser

Root-knot nematodes are plant parasites that damage the roots. During their infection process in roots, the plants can be attacked on leaves by insects, e.g. caterpillars. Interestingly, the caterpillars might arrive on and feed on the plants when the root nematode is at different stages of its infection cycle. We investigated how the caterpillar might affect root defence responses triggered by root nematode at different stages of its life cycle. We demonstrated that caterpillar feeding on the nematode-infected plants decreases the jasmonic acid signalling when the nematode is at the reproduction stage. More studies are needed to progress this understudied field.

## Introduction

Tomato is ranked the most consumed vegetable globally, with >170.8 million tons produced in 2017 alone (Omondi 2018; FAO 2019). This yield is ~30 % times more than a decade earlier (Oishimaya 2017). Like other crops, tomato plants experience high pest pressure by, e.g., nematodes, arthropods, bacterial and fungal pathogens. This pest pressure reduces the growth and limits tomato yield (Kumar *et al.* 2016; Garcia *et al.* 2018; van Dam *et al.* 2018). Root-knot nematodes (RKNs) are globally occurring, soil-borne pathogens that attack plants at their roots. The infective second-stage juveniles (J2s) hatch in the soil, where they locate and infect the roots of a susceptible host. Upon penetrating the roots, the J2s migrate intercellularly until they reach the vascular tissues. There they establish their permanent feeding sites (Niebel *et al.* 1994; Williamson and Gleason 2003; Gheysen and Mitchum 2011). Their infection impairs the translocation of water and minerals from the roots to the shoots, which can limit the plant’s productivity and fitness (Abad *et al.* 2008; Jones *et al.* 2013). At the same time, above-ground (AG) herbivores, such as leaf-chewing caterpillars, may be present on the plant. The leaf loss due to caterpillar feeding can adversely impact on primary plant processes, such as the rate of photosynthesis, which are directly related to the plant’s productivity (Meyer and Whitlow 1992; Mitchell *et al.* 2016). Together the damage caused by RKN and herbivorous insects can reduce crop production by ~20 % annually, making them agro-economically important crop pests (Karajeh 2008; van der Meijden 2015; Mitchell *et al.* 2016). Commonly, chemical pesticides are used to control crop pests, such as nematodes and insect herbivores. Although these pesticides might be effective, several of them are currently banned from use due to their detrimental effects on human health and the environment (Franco *et al.* 2015; Borel 2017). Efforts to identify natural plant resistance traits for AG and below-ground (BG) herbivores may help to develop sustainable pest management strategies.

Plants rely on constitutive and inducible defence responses to protect themselves against attackers. Constitutive responses are described as the physical barriers, such as thorns and trichomes, and chemical traits, such as alkaloids and glucosinolates, usually expressed independently of herbivore or pathogen attack (Wittstock and Gershenzon 2002). Induced defences are stimulated by herbivore feeding or pathogen attack, which results in the induction of specific plant phenotypic responses (Karban 2011; Boots and Best 2018). In addition, plants can tolerate herbivory via the re-allocation of resources to undamaged plant parts, followed by compensatory growth, or by increasing the rate of photosynthesis (Mauricio *et al.* 1997; Peterson *et al.* 1998; Retuerto *et al.* 2004; Boege *et al.* 2007; Núñez-Farfán *et al.* 2007; Fornoni 2011; Koch *et al.* 2016; Mitchell *et al.* 2016). These changes influence critical plant physiological processes and can adversely impact the performance of herbivores.

Plant hormonal signalling governs herbivore-induced defence responses. Among the many plant hormones described so far, jasmonic acid (JA), salicylic acid (SA), ethylene (ET) and abscisic acid (ABA) are the main signalling hormones that fine-tune plant defence responses upon attack (Pieterse *et al.* 2009, 2012). Interaction, or cross-talk, between phytohormonal pathways, results in specific defence responses, which tailor the defensive response to the particular attacker (Pieterse *et al.* 2009, 2012; Li *et al.* 2019). Induction of defence responses at the site of attack often results in systemic signalling to distal non-attacked plant parts, thereby protecting them against future attacks (Martínez-Medina *et al.* 2013; van Dam *et al.* 2018). Moreover, systemic-induced responses may influence the attraction, behaviour and performance of other organisms sharing the same host (Bruce 2014). As a consequence, induced responses play an essential role in indirect interactions between AG and BG herbivores feeding on the same plant (van Dam and Heil 2011).

Most studies investigating plant-mediated interactions between AG and BG herbivores focus on how AG herbivore-induced responses are affected by BG herbivory (Erb *et al.* 2009; Kumar *et al.* 2016; Arce *et al.* 2017; Hoysted *et al.* 2017; van Dam *et al.* 2018). Only a few studies analysed how AG-induced responses affect BG-feeding herbivores or pathogens. These studies report that AG herbivory induces systemic responses in the roots of crops (e.g., potato, tomato) and grass species (Kafle *et al.* 2017; Wang *et al.* 2017; Hoysted *et al.* 2018). Both primary and secondary metabolites play a role in plant-mediated interactions between AG and BG insect herbivores. For example, AG feeding by aphids changes potato root exudates by reducing amounts of glucose and fructose, which diminish cyst hatching (Hoysted *et al.* 2018). Defoliation by clipping increases nitrogen concentration in roots, which in return increases the total abundance of two species of root-feeding nematodes (Wang *et al.* 2017). Similarly, AG feeding by *Manduca sexta* on *Nicotiana attenuata* induces jasmonate-dependent facilitation of plant-parasitic nematode (PPN) abundance in the field, and RKN (*Meloidogyne incognita*) reproduction in a greenhouse (Machado *et al.* 2018). Collectively, these studies demonstrate that plant responses induced by AG herbivory can systemically affect BG defence responses.

The few studies available show that systemic-induced responses triggered by AG herbivory cause different effects on root feeders (Huang *et al.* 2013; Kafle *et al.* 2017; Wang *et al.* 2017; Hoysted *et al.* 2018; Machado *et al.* 2018). Partly the differences in the observed interaction outcomes are due to variation in the timing and sequence of arrival of both AG- and BG-feeding organisms (Erb *et al.* 2011; Kafle *et al.* 2017; Wang *et al.* 2017). In nature, root herbivores commonly colonize the plant before shoot herbivores arrive. This natural sequence of pest arrival follows from the fact that roots develop first (Bezemer and van Dam 2005). For PPNs, such as RKNs, these factors are particularly relevant. As obligate root feeders, RKNs undergo different distinct life cycle stages. In the *invasion stage*, J2s enter the root at the zone of elongation and move towards the vascular cylinder. Then they turn around and move several body lengths upwards before settling and initiating feeding (Robinson and Perry 2006). This movement occurs between the cells (intercellularly), which also reduces the elicitation of defence responses because only a few cells are damaged (Caillaud *et al.* 2008; Gheysen and Mitchum 2011). In the *establishment stage*, the juveniles become sedentary and inject various effectors to establish the so-called ‘giant cell’. This giant cell serves as their feeding site. The cells surrounding the giant cells undergo proliferation and enlargement, and, in due time, they become visible to the human eye as a gall or a ‘root-knot’ (Rodiuc *et al.* 2014; Escobar *et al.* 2015). We refer to this stage, in which the nematode establishes a feeding site, as the galling stage. At their feeding site, the nematodes acquire resources and develop through three molts to mature and reach the *reproduction stage*. The female nematode’s body swells up and becomes pear-shaped. When the eggs are ripe, the females release their eggs into the rhizosphere, and another cycle begins (Caillaud *et al.* 2008; Gheysen and Mitchum 2011). In each infection stage, the nematodes’ growth and development depend on the injection of different effectors into the host cells (Quentin *et al.* 2013; Favery *et al.* 2016; Gheysen and Mitchum 2019). These effectors trigger different hormonal signalling pathways, including JA, SA, ET and ABA (Caillaud *et al.* 2008; Kyndt *et al.* 2017; Gheysen and Mitchum 2019). Because hormones are generally involved in plant defence induction, systemic defence responses induced by AG herbivores might affect nematodes and the local responses they induce in the roots. Moreover, the effect that AG herbivores may have on BG defence signalling triggered by root herbivores may depend on the life cycle stage in which the nematodes are at the time point of AG attack.

Here, we used tomato (*Solanum lycopersicum* ‘Moneymaker’) and two generalist crop pests, the RKN *M. incognita* and larvae of *Spodoptera exigua*, as the study system to analyse the molecular mechanisms mediating interactions between AG herbivores and nematodes. Previous studies showed that interactions between RKN and shoot herbivores can be governed by JA-dependent responses, evidenced by changes in jasmonates levels in *N. attenuata* (Machado *et al.* 2018) and the production of trypsin protease inhibitors in tomato (Arce *et al.* 2017). These interactions may also involve cross-talk between hormonal pathways, such as JA–SA (van Dam *et al.* 2018) and JA–ABA (Erb *et al.* 2009; Kyndt *et al.* 2017). Therefore, we measured phytohormone concentrations (JA, SA, ABA) and the expression of several marker genes for hormonal signalling; *Lipoxygenase D* and *Leucine aminopeptidase A* (JA markers), *Le4* (ABA marker) and *Basic-β-1,3-glucanase (GluB)* (ET marker) in roots **[see**Supporting Information—Table S1**]**. Tomato is also known to produce steroidal glycoalkaloids, such as α-tomatine, as a defence to generalist herbivores (Friedman 2002; Cárdenas *et al.* 2015). Hence, we included measurements of α-tomatine and the expression of glycoalkaloid metabolism (GAME) genes *Jasmonate-responsive Ethylene Response Factor 4 (JRE4)* and *GAME1*. We specifically analysed how 24 h of AG feeding affected these defence-related traits in roots that were infected with *M. incognita* at 5, 15 and 30 days post-nematode inoculation (dpi). These time points coincide with the invasion (5 dpi), galling (15 dpi) and reproduction (30 dpi) stages of this nematode. With this approach, we aimed to assess whether the nature of the interaction between shoot- and root-induced responses depends on the developmental stage of the RKN.

## Materials and Methods

### Study plant, root and shoot organisms

In all our experiments, we used tomato (*S. lycopersicum* ‘Moneymaker’) as the model plant. The RKN *M. incognita* was used as root herbivore, and the larvae of the generalist herbivore *S. exigua* were used as shoot herbivores. We obtained *M. incognita* eggs from Rijk Zwaan (De Lier, The Netherlands) and maintained a glasshouse stock on tomato ‘Moneymaker’ for 8 weeks. Similar to a previous study (Martínez-Medina *et al.* 2017), we initiated the colony from a single egg mass, and 8 weeks later extracted eggs for use in the bioassay. We purchased *S. exigua* eggs from Entocare C.V. Biologische Gewasbescherming (Wageningen, The Netherlands) and maintained a laboratory colony on artificial diet, in a growth chamber set at 25 °C constant, 12-h photoperiod and 45 % relative humidity (RH).

### Plant growth condition and herbivores infection

The tomato seeds were obtained from Intratuin B.V (Woerden, The Netherlands). Before germination, the seeds were surface-sterilized by immersion in 40 mL of 10 % sodium hypochlorite solution for 4 min. Afterward, the seeds were rinsed four times with water. Each round of rinsing was for 10 min. The sterilized seeds were placed on moistened glass beads and allowed to germinate at 27 °C in the dark for 3 days, followed by 4 days in a plant growth chamber (CLF PlantClimatic, CLF PlantClimatics GmbH, Wertingen, Germany). The growth conditions were 16-h:8-h day:night cycle, 55 % RH and 60 % (65 μmol s^−1^ m^−2^) light intensity. One-week-old seedlings were transplanted into sterilized 1:1 sand:soil mixture in 11 × 11 × 12 cm pots. They were grown in a glasshouse at 26 ± 3 °C:23 ± 3 °C day:night, with 16-h:8-h light:dark and RH was maintained at ~30 %. The plants were watered as required and supplemented weekly with 50 % strength Hoagland solution. The plants were grown for three more weeks before using them in bioassays. We randomly selected healthy plants of similar size and appearance for our experimental treatments. We divided the plants into two groups; one group was inoculated with *M. incognita* eggs (3000 eggs per mL), and the other group was mock-inoculated with water. In the *M. incognita-*inoculated plants, we set three time points to coincide with the main nematode life cycle stages. These were 5 dpi (invasion stage), 15 dpi (galling stage) and 30 dpi (reproduction stage). At each of these time points, plants were subjected to four different treatments, each with 10 biological replicates. The treatments were control (plants without herbivores or nematodes); BG infection (plants challenged with *M. incognita*); AG herbivory (plants challenged with *S. exigua*); and both BG infection and AG herbivory (plant challenged with *M. incognita* in the roots followed by *S. exigua* feeding on leaves). We infested the plants assigned to leaf feeding with one second-instar *S. exigua* caterpillar. The *S. exigua* caterpillars were confined to a 7-cm (diameter) round clip cage placed on one fully expanded leaf close to the tip (see Fig. 4D in Bandoly and Steppuhn (2016)). In plants without shoot herbivory, an empty clip cage was mounted on a leaf at a similar position to the one used in plants with shoot herbivory. The *S. exigua* larvae were allowed to feed for 24 h. Other studies showed that this time period suffices to affect defence metabolites and genes in roots. For example, 24 h of AG herbivory by *M. sexta* and *Spodoptera littoralis* on *N. attenuata* results in systemic induction of JA-related genes expression in roots (Fragoso *et al.* 2014). After this time, we harvested the roots by gently removing them from the pots. The soil was removed by soaking the whole root into a bucket filled with tap water. Then the roots were rinsed with running tap water and dried with filter paper. After quickly counting the number of galls (especially for roots collected at the galling and reproduction stages) **[see**Supporting Information—Fig. S1**]**, the roots were wrapped in clean labelled aluminium foil, and immediately shock-frozen in liquid nitrogen. The root samples were stored at –80 °C, pending molecular and metabolite analyses.

### Quantitative reverse transcription-polymerase chain reaction analysis

Total RNA was extracted from ~100 mg fresh weight per root sample according to the method described by Oñate-Sánchez and Vicente-Carbajosa (2008). First-strand cDNA was synthesized from 1 µg DNase-free mRNA using Revert Aid H-minus RT (Thermo Fisher Scientific Baltic UAB, Vilnius, Lithuania) following the manufacturer’s instructions. Real-time qPCR reactions and relative quantification of specific mRNA levels were performed according to Martínez-Medina *et al.* (2017) by using a CFX 384 Real-Time PCR system (Bio-Rad Laboratories Inc., Singapore) and the gene-specific primers described in Supporting Information—Table S1. These genes were selected from previously published articles where their involvement in tomato biotic interactions is reported (Uppalapati *et al.* 2005; Martínez-Medina *et al.* 2013; Yan *et al.* 2013; Abdelkareem *et al.* 2017). The data were normalized using the housekeeping gene (*SIEF X14449*), which encodes for the tomato elongation factor-1α, a commonly used and stable reference gene for data normalization in studies on induced responses in tomato (Miranda *et al.* 2013; Martínez-Medina *et al.* 2017). Data were analysed by the 2^−∆∆ct^ method (Livak and Schmittgen 2001).

### Determination of phytohormone concentration

We extracted and quantified phytohormones following the protocol described by Machado *et al.* (2013). In brief, ~100 mg fresh weight per root sample was extracted with 1 mL ethyl acetate containing 40 ng of each of the following internal phytohormone standards: *D*_6_-JA and *D*_6_-SA, and *D*_6_-ABA. The extracts were vortexed for 10 min using a Thermomixer, then centrifuged at 15 000 × g, 4 °C for 20 min, the supernatants were transferred to a new tube and evaporated to dryness at room temperature using a SpeedVac (Labconco Co-operation, Kansas, MO, USA). Remaining pellets were resuspended in 200 µL methanol:water (70:30) using an ultrasonic bath for 5 min and centrifuged at 15 000 × g, 4 °C for 5 min. The supernatant was collected for phytohormone measurement using liquid chromatography (Bruker Advance UHPLC, Bremen, Germany) coupled to a mass spectrometer (Bruker Elite EvoQ Triple quadrupole, Bremen, Germany) (LC/MS EVOQ) (Schäfer *et al.* 2016). The separation was achieved on a Zorbax Eclipse XDB-C18 column (4.6 × 50 mm, 1.8 µm, 80 Å, Agilent technologies, Santa Clara, CA, USA). Mobile phase was composed of A (0.05 % (v/v) aqueous formic acid) and B (0.05 % (v/v) formic acid in 100 % acetonitrile). The following gradient was used: 0–0.5 min, 5 % B; 0.5–0.6 min, 5–50 % B; 0.6–2.5 min, 50–100 % B; 2.5–3.5 min, 100 % B; 3.5–3.55 min, 100–5 % B; 3.55–4.5 min, 5 % B at flow rate of 400 µL min^−1^. All solvents used were LC-MS grade. The column temperature was kept constant at 42 °C.

After separation, the compounds were nebulized by electron spray ionization in the negative mode using the following conditions: capillary voltage 4500 eV, cone gas 35 arbitrary units/350 °C, probe gas 60 arbitrary units/475 °C and nebulizing gas at 60 arbitrary units. The phytohormones were identified based on their retention time and the monitored mass to charge ratio (*m*/*z*) transition. The *m*/*z* ratio of the phytohormones of interest were; (*m*/*z*) 209.12 → 59.00 for JA; (*m*/*z*) 263.13 → 153.00 for ABA and (*m*/*z*) 137.02 → 93.00 for SA. Samples were analysed in a randomized sequence with acetonitrile samples in between as background controls. Data acquisition and processing were performed using the ‘MS data Review’ software (Bruker MS Workstation, version 8.2). Phytohormone levels were calculated based on the peak area of the corresponding internal standard and the amount of fresh mass of plant material (ng^−1^ mg^−1^ fresh weight).

### Determination of the root α*-*tomatine concentrations

We extracted ~100 mg fresh weight of each root sample in a 2-mL Eppendorf tube with 1 mL solution containing 25 % of acetate buffer (2.3 mL acetic acid, 3.41 mg ammonium acetate dissolved in 1 L of Milli pure water, pH 4.8) and 75 % methanol. Tubes with extracts were inverted for 10 s and thoroughly mixed via shaking using a grinding ball mill (MM400, Retsch GmbH, Leipzig, Germany) set at 30 Hz for 5 min. To remove the solid particles in the extracts, we centrifuged them at 15 000 × g for 15 min, and the supernatant transferred into a new 2-mL Eppendorf tube, and the pellet was re-extracted as above. We mixed the first and second supernatant and transferred 200 µL of the combined extracts into a 2-mL HPLC vial and added 800 µL of the extraction buffer to obtain a 1:5 dilution for each sample. The extracts were stored at −20 °C, pending further analysis. Metabolites were characterized by injecting 1 µL of the extracts in a UPLC (Dionex 3000, Thermo Scientific). The chromatograph was equipped with a C18 column (Acclaim TM RSLC 120), 2.1 × 150 mm external dimension, 2.2 µm particle size and 120 Å pore size. The column was kept at 40 °C. The mobile phases (LC-MS grade solvents) were composed of solvent A: 0.05 % (v/v) aqueous formic acid and solvent B: 0.05 % (v/v) formic acid in acetonitrile. The multi-step gradient for solvent B was; 0–1 min 5 %, 1–4 min 28 %, 4–10 min 36 %, 10–12 min 95 %, 12–14 min 95 %, 14–16 min 5 %, 16–18 min 5 %. The flow was set to 400 µL min^−1^. We detected compounds using a maXis impact HD MS-qToF (Bruker Daltonics). Data were acquired in positive mode. Electron Spray Ionisation ion source conditions were; endplate offset = 500 V, capillary = 4500 V, nebulizer = 2.5 bar, dry gas = 11 L min^−1^, dry temperature = 220 °C. Transfer line conditions were: funnels 1 and 2 = RF 300 Vpp, isCID energy = 0 eV, hexapole = 60 Vpp, quadrupole ion energy = 5 eV, low mass = 50 *m*/*z*, collision cell energy = 10 eV, collision RF 500 Vpp, transfer time = 60 µs, pre-pulse storage = 5 µs. The mass spectrometer operated with a mass range of 50–1500 *m*/z and a spectral acquisition rate of 3 Hz. Sodium formate clusters (10 mM) were used for calibrating the *m*/*z* values. These sodium formate clusters were a mix consisting of 250 mL isopropanol, 1 mL formic acid, 5 mL 1 M NaOH and the final volume was adjusted to 500 mL. All analyses had a quality control sample, which was a pool of all the different experimental groups and time points. The quality control sample was analysed at the beginning and the end of the batch and after every 10 injections. The raw data files were processed using the program Compass DataAnalysis (Bruker Daltonics). The processing involved obtaining the extracted ion chromatogram (EIC) for a fragment of α*-*tomatine at the *m*/*z* value 578.4050 and *m*/*z* tolerance of ±0.1. We selected the option compound list to automatically calculate the peak areas of each EIC per sample per study time point. All the peak areas for α-tomatine were tabulated and used for multivariate statistical analysis.

### Statistical analysis

We created two data sets combining (i) phytohormone and α-tomatine levels, and (ii) defence markers and glycoalkaloid metabolism genes. In each combined data set, we tested the effects of *M. incognita* (Mi; with vs. without), and *S. exigua* (Se; with vs. without), and their interactions on the defence variables (i.e. the plant defence traits; phytohormone, α-tomatine and marker genes). Each data set was analysed using the permutational multivariate analysis of variance (PERMANOVA). Permutational multivariate analysis of variance was chosen because our data lacked homogeneity of variance or normal distribution; PERMANOVA does not require this because it uses a distribution-free permutation approach to partition the variance among treatments (Anderson 2017). The PERMANOVA analysis was run for each data set using the Adonis function, with the Gower dissimilarities method among samples, and 999 permutations in R v 3.6.1 software (R Core Development Team 2019). Where the PERMANOVA output showed significant effects for either factor or their interaction **[see**Supporting Information—Tables S2**and**S4**]**, we performed separate factorial linear model ANOVAs on each dependent variable, with *M. incognita* and *S. exigua* and their interaction as fixed factors. Once the main effect significantly affected any of the dependent variables, the differences among the four experimental treatments were tested using Tukey’s Honest Significant Difference test for multiple comparisons.

## Results

### Root infection by *M. incognita* alone affects the expression of root-inducible defences at different life cycle stages

**Figure 1.**
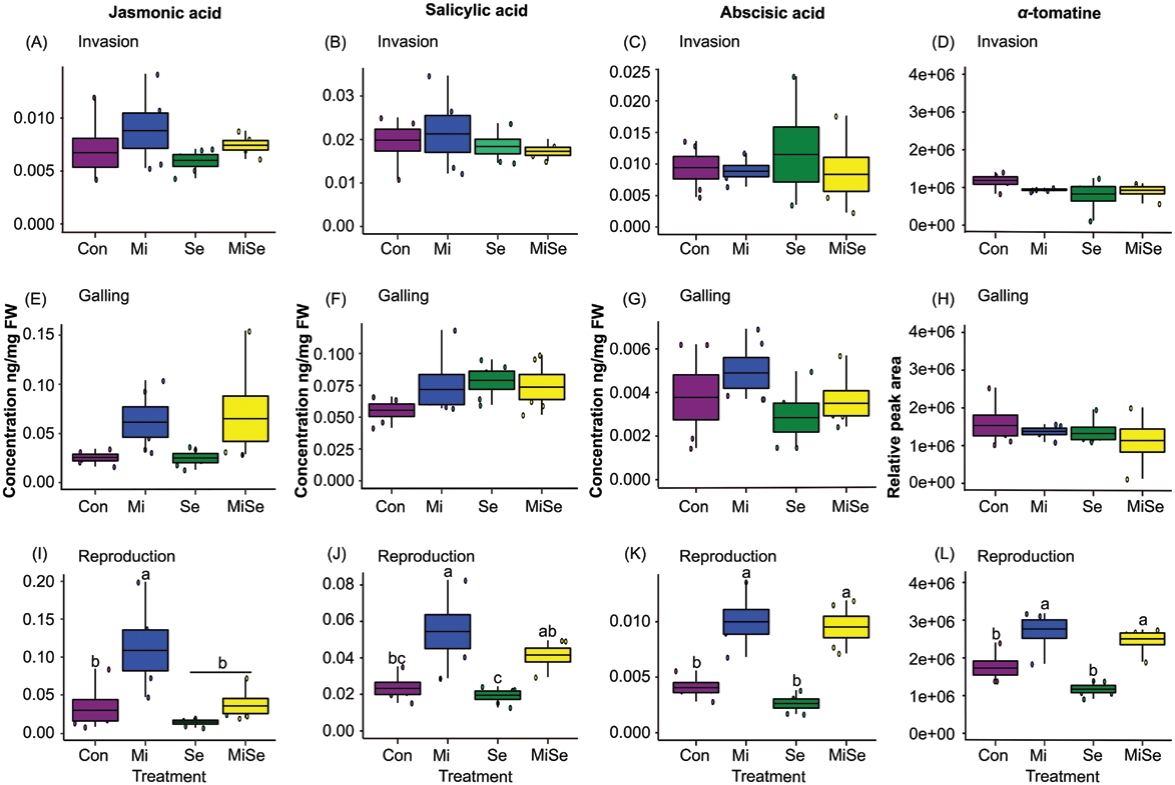
Phytohormone concentrations and relative peak area of α-tomatine. Mean concentrations (ng mg^−1^ fresh weight) of phytohormones and the relative peak area of α*-*tomatine in tomato plants infected with *Meloidogyne incognita* (Mi), infested with *Spodoptera exigua* (Se) or both (MiSe). Con = plant without herbivory. Box plots are the mean (±SEM) of jasmonic acid (A, E, I); salicylic acid (B, F, J); abscisic acid (C, G, K); α-tomatine (D, H, L) per treatment (*n* = 5) measured at the nematodes’ invasion (A–D), galling (E–H) and reproduction (I–L) stages. Different lower-case letters above the box plots indicate significant differences in mean values between treatments, determined via multiple comparisons Tukey’s HSD test after ANOVA at *P* ≤ 0.05.

**Figure 2.**
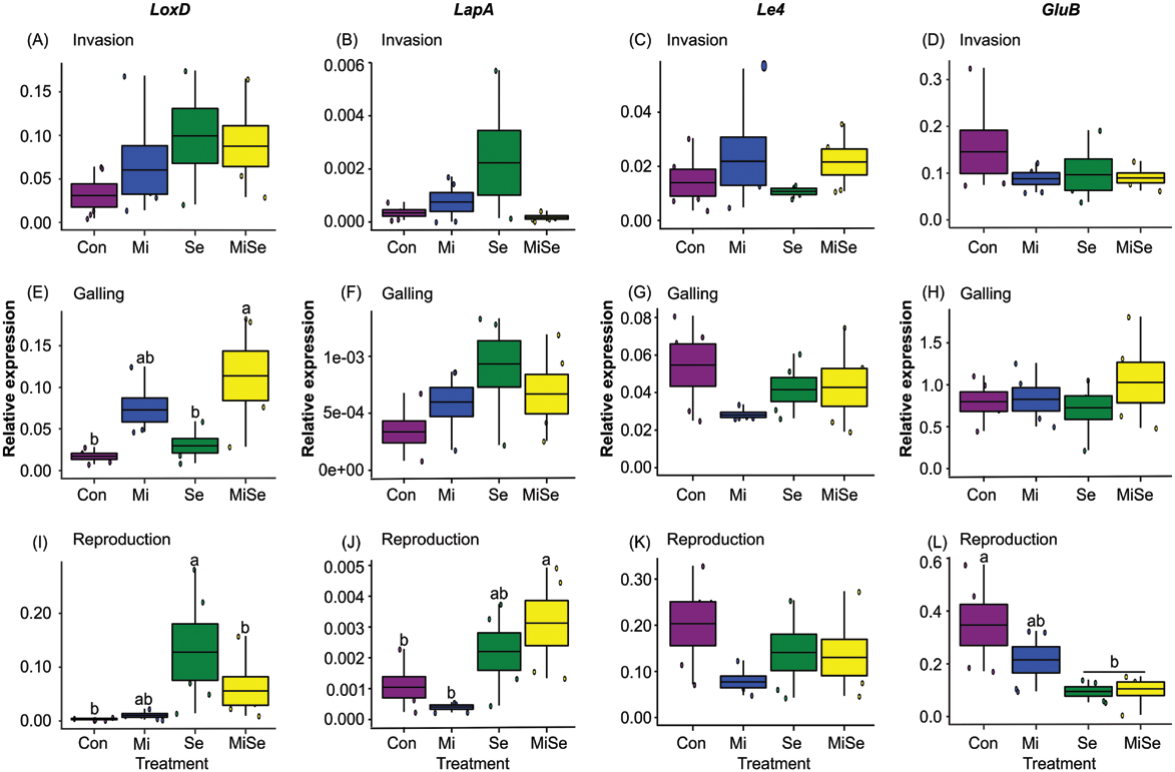
Expression of defence marker genes. Relative expression of defence marker genes in tomato plants infected with *Meloidogyne incognita* (Mi), infested with *Spodoptera exigua* (Se) or both (MiSe). Con = plants without herbivory. Expression values are normalized over the expression of the housekeeping gene (*SIEF X14449*) encoding for tomato elongation factor-1α. Box plots are mean (±SEM) expression values of *Lipoxygenase D* (*LoxD*); *Leucine aminopeptidase A* (*LapA*); *abscisic acid-responsive Le4* (*Le4*); *Basic-β-1-glucanase* (*GluB*) per treatment (*n* = 5) measured at the nematodes’ invasion (A–D), galling (E–H) and reproduction (I–L) stages, respectively. Different lower-case letters above the box plots indicate significant differences in mean expression among treatments, determined via multiple comparisons Tukey’s HSD test after ANOVA at *P* ≤ 0.05.

**Figure 3.**
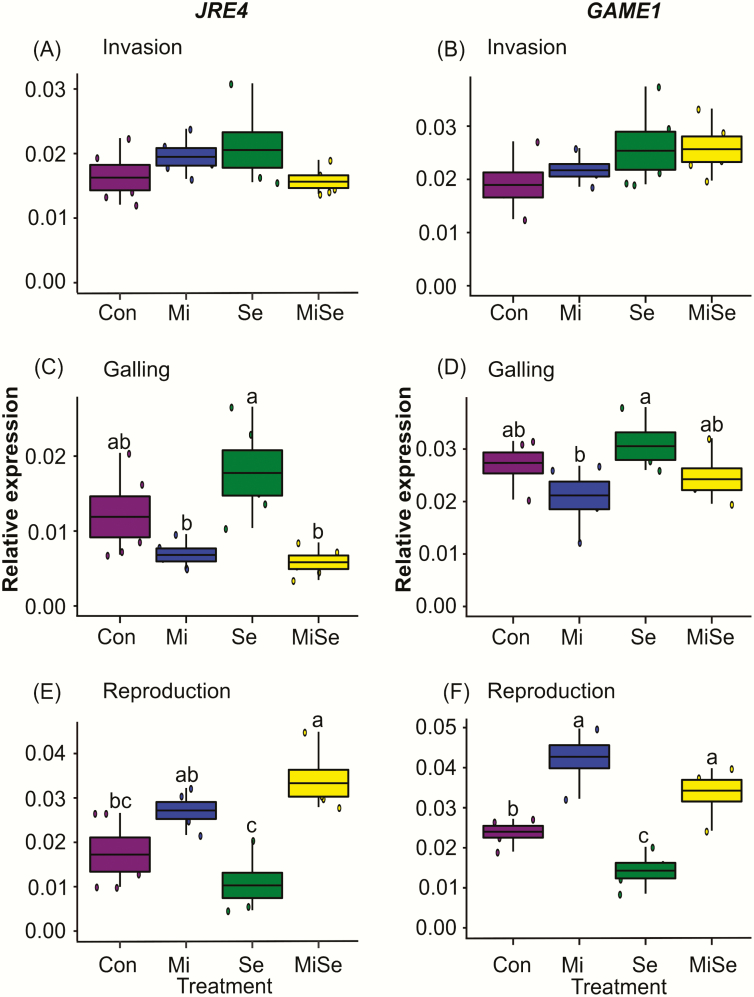
Expression of steroidal glycoalkaloid metabolism genes. Relative expression of steroidal glycoalkaloid metabolism genes in tomato plants infected with *Meloidogyne incognita* (Mi), infested with *Spodoptera exigua* (Se) or both (MiSe). Con = plants without herbivory. Expression values are normalized over the expression of the housekeeping gene (*SIEF X14449*) encoding for tomato elongation factor-1α. Box plots are mean (±SEM) expression values of *jasmonate-responsive ETHYLENE RESPONSE FACTOR 4* (*JRE4*; A, C, E); and *glycoalkaloid metabolism 1* (*GAME1*; B, D, F) per treatment (*n* = 5) measured at the nematodes’ invasion (A and B), galling (C and D) and reproduction (E and F) stages, respectively. Different lower-case letters above the box plots indicate significant differences in mean expression among treatments, determined via multiple comparisons Tukey’s HSD test after ANOVA at *P* ≤ 0.05.

We first considered how the nematode affected root-inducible defences at the invasion, galling and reproduction stages. We found that *M. incognita* root infection enhanced the induction of JA, SA, ABA and α-tomatine progressively during the infection process. In particular, the JA response in *M. incognita*-infected plants became more pronounced with the progression of the nematode’s life cycle compared to controls (Fig. 1A, E and I, blue box plots). At both the invasion and galling stages, the levels of these signalling hormones were increased, but only at the reproduction stage, the increases became significant compared to control plants (Fig. 1I–L, blue box plots; **see**Supporting Information—Table S3). In contrast, root infection by *M. incognita* did not trigger changes in the expression of the defence marker genes. We found that the expression of *LoxD*, *LapA*, *Le4*, *GluB* (Fig. 2, blue box plots; **see**Supporting Information—Table S5) and *JRE4* (Fig. 3, blue box plots; **see**Supporting Information—Table S5) remained similar to those observed in control plants regardless of the nematodes’ root infection stage. However, we observed significant upregulation in the expression of the *GAME1* transcripts at the nematodes’ reproduction stage compared to control plants (Fig. 3F, blue box plot; **see**Supporting Information—Table S5). The increase in *GAME1* transcripts correlated with an increase in α-tomatine concentrations in nematode-infected roots at the reproduction stage (Fig. 1L, blue box plot).

### The impact of *S. exigua* feeding on root defence responses in tomato plants depends on plant age

Next, we analysed the impact of *S. exigua* leaf herbivory on root defences of tomato plants without nematode infection. Due to the experimental set-up, which was designed based on the life stages of the nematodes, the plants that received only caterpillars were 4.8 (coinciding with the invasion stage), 6.2 (coinciding with the galling stage) and 8 (coinciding with reproduction stage) weeks old. We found that *S. exigua* leaf herbivory did not affect the levels of JA, SA, ABA and α-tomatine in tomato roots compared to the control plants, regardless of plant age (Fig. 1, green box plots; **see**Supporting Information—Table S3). In contrast, *S. exigua* herbivory triggered differential effects on the expression of the hormonal signalling and GAME marker genes (Figs 2 and 3, green box plots; **see**Supporting Information—Table S5). In the 4.8 (invasion stage) and 6.2 (galling stage) weeks old plants, the expression of the marker genes was not significantly different from controls (Figs 2A–H and 3A–D, green box plots; **see**Supporting Information—Table S5). Notably, when the plants were 8 weeks old, which coincided with the nematodes’ reproduction stage, the defence gene *LoxD* was upregulated compared to controls (Fig. 2I, green box plot; **see**Supporting Information—Table S5). The *LapA* and *Le4* expression levels were not significantly different compared to controls (Fig. 2J and K, green box plots; **see**Supporting Information—Table S5), while *GluB* was significantly downregulated compared to controls (Fig. 2L, green box plot; **see**Supporting Information—Table S5). The GAME gene *JRE4* was not affected while the *GAME1* was significantly downregulated compared to controls (Fig. 3E and F, green box plots; **see**Supporting Information—Table S5).

### Effects of *S. exigua* on *M. incognita*-induced responses depend on the nematodes’ infection stage

Because our primary interest was to analyse the effect of *S. exigua* AG feeding on root responses induced by *M. incognita* at different infection stages, we primarily focused on the comparison between *M. incognita*-infected plants (Mi treatment, blue box plots, Figs 1–3) with the double-infected plants (MiSe treatment, yellow box plots, Figs 1–3). We found that *S. exigua* herbivory on *M. incognita-*infected plants did not change JA levels at the invasion and galling stages compared to plants challenged with *M. incognita* alone (Fig. 1A and E, yellow box plots; **see**Supporting Information—Table S3). *Spodoptera exigua* herbivory on the *M. incognita*-infected plants significantly decreased the JA levels at the nematodes’ reproduction stage compared to plants infected with *M. incognita* alone (Fig. 1I, yellow box plot; **see**Supporting Information—Table S3). *Spodoptera exigua* feeding on *M. incognita*-infected plants did not affect SA, ABA and α-tomatine concentrations compared to plants challenged with *M. incognita* alone, regardless of the nematodes’ infection stage (Fig. 1B–D, F–H and J–L, yellow box plots; **see**Supporting Information—Table S3). Overall, we observed that the local nematode-induced responses dominated the nature of SA, ABA and glycoalkaloid responses in roots (Fig. 1; **see**Supporting Information—Table S3, main Mi effects). Similarly, *S. exigua* herbivory on *M. incognita*-infected plants triggered changes in the expression of marker genes depending on the nematodes’ root infection stages. At the invasion stage, the expression levels of both defence and GAME genes in double-infected plants were similar to those with *M. incognita* infection alone (Figs 2A–D, and 3A and B, yellow box plots; **see**Supporting Information—Table S5). At the galling stage, the JA biosynthesis marker *LoxD* overall increased in plants infected with *M. incognita* (**see**Supporting Information—Table S5, main Mi effect). Above-ground damage by *S. exigua* did not significantly alter this. A similar pattern was found for the expression levels of the other marker genes in plants with *M. incognita* and *S. exigua*; in the invasion and galling stage their expression levels were similar to plants with *M. incognita* infection alone (Figs 2E–H, and 3C and D, yellow vs. blue box plots; **see**Supporting Information—Table S5). During the reproduction stage, *S. exigua* herbivory on *M. incognita*-infected plants significantly upregulated *LapA* (Fig. 2J, yellow box plot; **see**Supporting Information—Table S5), whereas it had no significant effect on the other marker genes compared to plants infected with *M. incognita* alone (Figs 2I, K and L, and 3E and F, yellow box plots; **see**Supporting Information—Table S5). By comparing the double-infected plants to control plants and those infected with *S. exigua* only, it became clear that the downregulation of *GluB* by *S. exigua* (Fig. 2L; **see**Supporting Information—Table S5) is not affected by *M. incognita* infection. On the other hand, the significant main effects of *M. incognita* on the expression of *JRE4* and *GAME1* at the galling and reproduction stages were not changed by *S. exigua* feeding (Fig. 3C and D, and E and F, blue and yellow box plots; **see**Supporting Information—Table S5). Therefore, our results collectively suggest that *S. exigua* can affect nematode-induced root responses, in particular via the JA-signalling pathway, depending on the nematodes’ infection stage.

## Discussion

**Figure 4.**
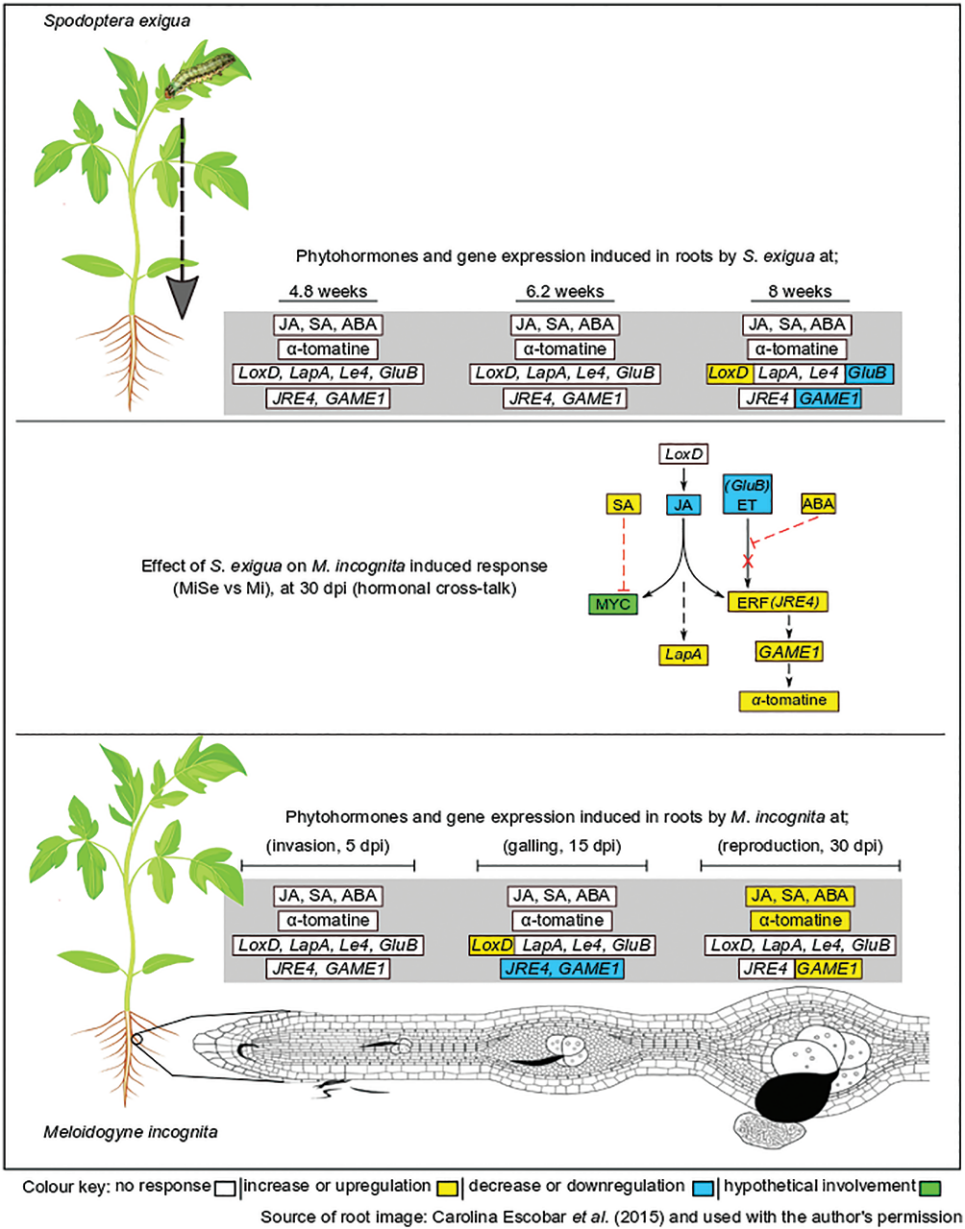
Interactions between root defence responses upon root and leaf herbivory. Schematic illustration of induced defences in tomato roots including the phytohormones jasmonic acid (JA), salicylic acid (SA), abscisic acid (ABA), the glycoalkaloid α*-*tomatine and defence genes (*Lipoxygenase D* (*LoxD*), *Leucine aminopeptidase A* (*LapA*), *Le4 abscisic acid-responsive*, *Basic-β-1-glucanase* (*GluB*)) and glycoalkaloid metabolism (GAME) genes (*jasmonate-responsive ETHYLENE RESPONSE FACTOR 4 transcription factor* (*JRE4*) and *GLYCOALKLOID METABOLISM 1* (*GAME1*)). The top panel represents phytohormones and gene expression induced in tomato roots by the caterpillar *Spodoptera exigua* on plants of different ages (4.8, 6.2 and 8 weeks). The bottom panel represents phytohormones and gene expression induced in tomato roots by the root-knot nematode (RKN) *Meloidogyne incognita* at different root infection cycle stages (invasion stage estimated at 5 days post-nematode inoculation (dpi), galling stage estimated at 15 dpi and reproduction stage estimated at 30 dpi). The middle panel shows the effect of *S. exigua* leaf feeding on root responses induced by *M. incognita* (MiSe) compared to those infected with *M. incognita* (Mi) alone at 30 dpi (hormonal cross-talk). White boxes: no response, yellow boxes: increase in trait levels or upregulation of gene expression, blue boxes: decrease in traits levels or downregulation of gene expression and green box: hypothetical involvement. In the proposed hormonal cross-talk schedule in the middle, dotted red lines show negative cross-talk, the black arrows show the steps in the JA pathway and the dashed black arrows represents several unknown steps. In our cross-talk model, we propose that the increase in SA affects the JA pathway negatively at the level of the MYC transcription factor. At the same time, the increase in ABA levels blocks the ethylene (ET) pathway, which regulates the ETHYLENE RESPONSIVE FACTOR (ERF) branch of the JA pathway. We hypothesize that the absence of ET promotes the activity of the *JRE4* transcription factor, which enhances transcription of the GAME pathway. Based on the response of the defence marker gene *LapA* in MiSe plants at 30 dpi, we also hypothesize that this pathway leading to late JA responses is involved in the interaction.

The goal of our study was to determine whether the impact of AG feeding on root defence responses induced by *M. incognita* depends on the nematodes’ life cycle. We tested this by exposing *S. exigua* caterpillars to tomato plants infected by *M. incognita* at different stages of the root infection cycle. We found that *S. exigua* affected *M. incognita* root-induced responses, mainly at the nematodes’ reproduction stage. In particular, the JA-signalling pathway was affected, as evidenced by lowered levels of JA in double-infected plants compared to plants infected with *M. incognita* alone. Jasmonic acid is known to regulate the GAME pathway in tomato via the *JRE4* transcription factor (Thagun *et al.* 2016). In this study, the attenuation of the JA pathway did neither lower α*-*tomatine concentrations nor the expression of the GAME genes (*JRE4* and *GAME1*) in double-infected plants compared to plants challenged with *M. incognita* alone (Fig. 4). This may be because the glycoalkaloid biosynthesis transcriptional coordinator *JRE4* can act downstream of JA signalling (Abdelkareem *et al.* 2017). Caterpillar feeding also enhanced *LapA* expression in double-infected plants at the nematodes’ reproduction stage compared to plants challenged with *M. incognita* alone. *LapA* acts downstream of JA signalling as a modulator of late wound-induced responses (Fowler *et al.* 2009). *LapA* expression is induced by external application of ABA, methyl jasmonate (MeJA) and ET (Chao *et al.* 1999). Here, the levels of ABA in double-infected plants remained elevated, which could be related to the upregulation in *LapA* expression. Cross-talk between phytohormones is widely recognized as a mechanism to tailor herbivore-induced responses to specific combinations of attackers (Pieterse *et al.* 2009; Zamioudis and Pieterse 2012). Cross-talk between the JA-signalling pathway and both the SA and ABA pathways may also explain why glycoalkaloid levels remained increased in double-infected plants at the nematodes’ reproduction stage compared to plants infected with *M. incognita* alone, despite lowered JA levels, *GluB* expression and no effect on *LoxD* expression compared to *M. incognita*-infected roots (Fig. 4). This cross-talk of SA and ABA with the JA pathway might also occur downstream of JA biosynthesis, e.g. at the level of transcription factors like MYC or ERF and in our case, *JRE4* (Fig. 4).

To date, the elicitation of root defences by endoparasitic nematode infection at later time points in their life cycle is virtually undescribed; most papers focus on signalling events occurring at 1–7 days after infection (Kyndt *et al.* 2012a, b; Kammerhofer *et al.* 2015; Martínez-Medina *et al.* 2017). Here, we found that *M. incognita* infection at the invasion and galling stages did not elicit strong defence responses, either on the level of phytohormones, gene expression or glycoalkaloid production. The lack of significant defence induction during the invasion stage can be partly attributed to how the RKNs migrate inside the roots. Once the J2s of RKN are inside roots, they avoid damaging plant cells by moving intercellularly through soft tissues of the host plant root tissues (Gheysen and Mitchum 2011; Gheysen and Jones 2013). Also, RKNs secrete effector proteins that play an essential role during both the penetration (invasion) and the establishment and galling phases. These effectors suppress host defence responses and help the nematode to establish a permanent feeding site (Abad and Williamson 2010; Mitchum *et al.* 2013). For instance, the rice pathogenic nematodes *M. graminicola* and *M. javanica* excrete the effectors, *Mg-MSP18* and *Mj-MSP18*, between 7 and 21 dpi to suppress the activation of their host’s immune responses, such as the hypersensitive response (Grossi-de-Sa *et al.* 2019). In our study, *M. incognita* did not induce significant root defences at the galling stage. We correlate this lack of defence induction to the fact that *M. incognita* utilizes effector proteins to repress plant responses in roots during the galling stage. For example, when feeding on *A. thaliana, M. incognita* secretes the effector *Mi-CTR* into the roots. This lowers pathogen-associated molecular pattern (PAMP)-triggered immunity (PTI) by suppressing the transcription of defense genes, such as *WRKY33,29, PDF1.2* and *pathogen related protein-1 (PR1)* (Jaouannet *et al.* 2013). The effect of *Mi-CTR* occurs after root invasion and initiation of the giant cells 21 dpi most likely to ensure successful establishment (Jaouannet *et al.* 2013).

Interestingly, when *M. incognita* reached the reproduction stage, we observed an induction of defence responses. We found that *M. incognita* infection increased all phytohormone levels measured, as well as the concentration of α*-*tomatine and the expression of its biosynthesis gene *GAME1*. Possibly, the swelling of the female bodies with the ripening eggs intensifies the cell damage at the feeding sites, leading to the observed hormonal and defence responses. It is remarkable, however, that the expression patterns of defence-signalling marker genes are not affected in the same way. Possibly the expression of defence marker genes might be regulated by effector proteins that are only secreted by female RKN during the reproduction stage. For instance, the *Misp12* effector is specific to *M. incognita* and secreted by mature females at least 28 dpi (Xie *et al.* 2016). Overexpression of *Misp12* suppresses *PR1* and *phenylalanine ammonia-lyase-5 (PAL5)* genes (SA pathway markers) in *N. benthamiana.* In *Misp12*-silenced plants, an upregulation of the *proteinase inhibitor 2 (Pin2)* (JA pathway marker) is reported. The authors suggest that *Misp12* might be involved in the maintenance of giant cells during the reproduction stages (Xie *et al.* 2016).

The systemic effect of *S. exigua* feeding on root hormone levels and defence responses was much less pronounced than local nematode-induced responses. On the one hand, this may be because the caterpillars fed only for 24 h on the plant, while the nematodes were continuously feeding. In other studies, shoot feeding by herbivores, including *S. exigua* and *Pieris rapae*, was applied for 2–7 days before defence responses were observed in the roots (Danner *et al.* 2015; Kafle *et al.* 2017). Possibly, 24 h of AG feeding may have been too short to elicit strong systemic responses in tomato roots. Moreover, systemic responses are generally weaker than locally induced responses (van Dam *et al.* 2001; Babst *et al.* 2009; Ádám *et al.* 2018). For example, leaf feeding by diamondback moth caterpillars in *Brassica oleracea* elicited slight systemic JA responses in the roots compared to the local induction by *Delia radicum* (Karssemeijer *et al.* 2020). In another study, shoot feeding by *P. rapae* larvae on *B. rapa* plants elicits much lower root volatile emissions than local damage by *D. radicum* larvae (Danner *et al.* 2015).

Interestingly, we found that the age of the plant affects the systemic response as well. In our experimental set-up, we applied nematode eggs at one single time point. Consequently, the *S. exigua* caterpillars were placed on tomato plants that were at different ages and ontogenetic stages. The expression of some defence marker genes was significantly upregulated (Fig. 2I) or downregulated (Fig. 3F) by *S. exigua* feeding only in the last batch of plants, which were 8 weeks old and flowering. It has been reported that herbivore-induced plant responses can significantly change as a function of plant ontogenetic stage (Quintero and Bowers 2011, 2012). For instance, the concentration of iridoid glycosides in *Plantago lanceolata* roots after AG herbivory was twice as high in mature plants compared to young plants (Quintero and Bowers 2011).

In nature, plants are likely to interact with AG herbivores and RKN at the same time. Here we found that *S. exigua* herbivory differentially affects the root-induced responses by *M. incognita* in tomato roots. These effects occurred in dependence on the life cycle of the nematode, whereby the impact was the strongest in the reproductive stages. Herbivore identity and sequence of arrival on the target host plant are some of the critical factors shaping interactions between AG–BG herbivores (Erb *et al.* 2011; Sarmento *et al.* 2011; Kafle *et al.* 2017). We conducted our experiment by first infecting the plants with RKN. This is likely the natural sequence of arrival because the roots develop before the shoots after seed germination. Moreover, nematodes are ubiquitous in natural systems. Roots are therefore likely to be invaded with nematodes before herbivores arrive on AG organs (Hoysted *et al.* 2018; van Dam *et al.* 2018). *Spodoptera exigua* feeding on *M. incognita*-infected plants reduced JA but not SA concentrations. In a similar study, *M. incognita* were allowed to colonize tomato plants that had experienced 7 days of *S. exigua* feeding, followed by a lag phase of another 7 days (Kafle *et al.* 2017). The authors found that after 14 days of *M. incognita* infection, the root JA levels decreased in tomato plants that were previously damaged by *S. exigua.* Combining our results with this study, we conclude that it may not matter whether the nematode or the AG herbivore infects first; AG feeding seems always to reduce RKN-induced JA levels in the roots.

Jasmonates are essential regulators of systemic signalling between AG and BG tissues (Wasternack 2007; Wasternack and Hause 2013). It has been established that JAs regulate the steroidal glycoalkaloid metabolism pathway via the *JRE4* transcription factor (De Geyter *et al.* 2012; Cárdenas *et al.* 2016; Thagun *et al.* 2016). Here the expression of *JRE4* was not altered by *S. exigua* feeding alone, nor did the caterpillar alter the *M. incognita*-induced upregulation of this transcription factor. Notably, the expression of *LapA* (JA marker) was significantly upregulated in double-infected plants compared to plants infected with *M. incognita* only, while *LoxD* expression was similar when *S. exigua* co-occurred with *M. incognita.* Our results suggest that the interaction between *M. incognita* and *S. exigua* might rely on the induction of late wounding responses regulated by *LapA* downstream of JA synthesis, e.g., on transcription factor level (Fig. 4). Unfortunately, our experimental set-up did not allow us to precisely determine the role of *LapA* because the plants with RKN in different life cycle stages also differed in age. *LapA* might also be associated with plant development, especially in the flowering stage, as reported by Chao *et al.* (1999).

Finally, the induction of JA levels by *M. incognita* infection was accompanied by an increase in α-tomatine production. Increases in JA and α*-*tomatine concentrations upon nematode attack or exogenous application of elicitors, such as MeJA, have been reported in tomato and other plant species (Abdelkareem *et al.* 2017; Kafle *et al.* 2017). Glycoalkaloids are usually associated with increased generalist herbivore resistance (Ökmen *et al.* 2013; Abdelkareem *et al.* 2017). In our study, we did not measure the ecological consequences associated with these defence responses, e.g., for later arriving herbivores. Further studies to test the effects of α-tomatine on the performance of the RKNs may reveal their effectiveness as defences against this generalist herbivore.

## Conclusions

Our study examined the impact of AG chewing herbivores on root-induced responses by RKN at different life cycle stages. We found that both the AG chewing herbivore and the RKN affect root defences. The effect of root infection by RKN alone, as well as the effect of AG herbivory on RKN-induced root defence responses, depends on the nematode’s life cycle stage. Studies testing the impact of long periods of AG herbivory on nematode-induced root responses are needed to reveal how the interactions with BG responses might change over longer interaction times. Such studies will help to optimize tomato breeding efforts towards cultivars with high resistance to AG and BG insect pests and pathogens.

## Supporting Information

The following additional information is available in the online version of this article—

Figure S1. The number of root galls counted in tomato plants roots upon root infection by *Meloidogyne incognita.*

Table S1. List of primers sequences used for quantitative polymerase chain reaction (qPCR).

Table S2. Permutational multivariate analysis of variance (PERMANOVA) results based on Gower dissimilarities on phytohormone and α*-*tomatine data for herbivory effects of *Meloidogyne incognita* root-knot nematodes (RKNs) and the caterpillars of *Spodoptera exigua*.

Table S3. Two-way factorial analysis of variance (ANOVA) results on phytohormone and α*-*tomatine for the herbivory effects of *Meloidogyne incognita* root-knot nematode (RKN) and the caterpillars of *Spodoptera exigua*.

Table S4. Permutational multivariate analysis of variance (PERMANOVA) results based on Gower dissimilarities on gene expression data for herbivory effects of *Meloidogyne incognita* root-knot nematode (RKN) and the caterpillars of *Spodoptera exigua*.

Table S5. Two-way factorial analysis of variance (ANOVA) results on gene expression for the herbivory effect of *Meloidogyne incognita* root-knot nematode (RKN) and the caterpillars *of Spodoptera exigua.*

plaa029_suppl_Supporting_Information

## Data Availability

The data underlying this study are published as open access at the iDiv Data Repository (Mbaluto *et al.* 2020; http://idata.idiv.de/ddm/Data/ShowData/1816).
