# Supplementary material for: The impact of Spodoptera exigua herbivory on Meloidogyne incognita-induced root responses depends on the nematodes’ life cycle stages
Source: AoB Plants. 2020 Jun 24;12(4):plaa029. doi: 10.1093/aobpla/plaa029 (PMC7336558; doi:10.1093/aobpla/plaa029)
Supplement: plaa029_suppl_Supporting_Information [file plaa029_suppl_supporting_information.pdf]

The impact of *Spodoptera exigua* herbivory on *Meloidogyne incognita* induced root responses depends on the nematodes' life cycle stages

Crispus M. Mbaluto[1,4], Esraa M. Ahmad[2], Melody Fu[3], Ainhoa Martínez-Medina[1,5], Nicole M. van Dam[1,4*]

Supporting Information

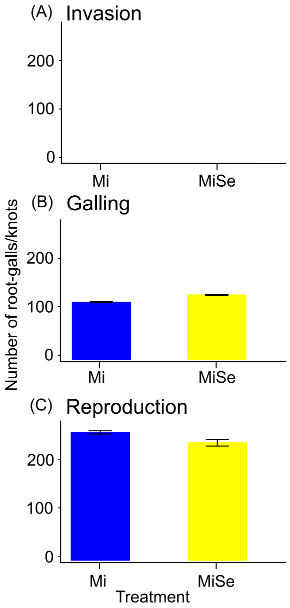

Figure S1 the number of root galls counted in tomato plants roots upon root infection by *Meloidogyne incognita.* Average number of root galls counted in tomato roots infected with *Meloidogyne incognita* (Mi) or co-infected with both shoot herbivore *Spodoptera exigua* (Se) and *M. incognita* (MiSe). Roots were harvested at the nematodes' invasion (A), galling (B), and reproduction (C) stages. Data are the mean ± standard error (*n*=10).

Table S1 List of primers sequences used for qPCR

Target gene	Hormone pathway	Primer orientation	Primer sequences (5'-3')
Lipoxygenase D (LoxD)[a]	Jasmonic acid (JA)	Forward<br>Reverse	GGCTTTATTTCACACAGAGATA<br>ATGTGCTGCCAATATAAATGGTTCC
Leucine aminopeptidase (LapA)[b]		Forward<br>Reverse	ATCTCAGGTTTCCTGGTGGAAGGA<br>AGTTGCTATGGCAGAGGCAGAG
Basic-β-1,3-glucanase (GluB)[c]	Ethylene (ET)	Forward<br>Reverse	CCATCACAGGGTTCATTTAGG<br>CCATCCACTCTCTGACACAACT
Abscisic acid-responsive (Le4)[c]	Abscisic acid (ABA)	Forward<br>Reverse	ACTCAAGGCATGGGTACTGG<br>CCTTCTTTCTCCTCCCACCT
Jasmonic-responsive ETHYLENE RESPONSE FACTOR (ERF) transcription factor (JRE4)[d]	coordinate transcription of metabolic genes in steroidal glycoalkaloids biosynthesis	Forward<br>Reverse	TGTTTCCTCCGGTGTTACGG<br>CGATTTTTTCGAAACTCTTTCC
GLYCOALKALOID METABOLISM 1 (GAME1)[d]	convert acetyl-coenzyme A (*acetyl*-CoA) to steroidal glycoalkaloids	Forward<br>Reverse	TTGCCGGATGTTCCATGATCG<br>CTAATGAAGAAACAGCGTCCTGG
SIEF X14449 (Housekeeping gene)[c]	Elongation factor-1α	Forward<br>Reverse	GATTGGTGGTATTGGAACTGTC<br>AGCTTCGTGGTGCATCTC

[a]Uppalapati *et al.* (2005); [b]Yan *et al.* (2013); [c]Martínez-Medina *et al.* (2013); [d]Abdelkareem *et al.* (2017)

Table S2 Permutational Multiple Analysis of Variance (PERMANOVA) results based on Gower dissimilarities on phytohormone and *α*-tomatine data for herbivory effects of *Meloidogyne incognita* root-knot nematodes (RKN) and the caterpillars of *Spodoptera exigua*. Root samples were collected from tomato plants infected with *M. incognita* at different life cycle stages. At the same time, root materials were collected from control and *S. exigua* infested plants. The single and interactive effects of *M. incognita* and *S. exigua* feeding on jasmonic acid, salicylic acid, and abscisic acid were determined via a MANOVA model with *M. incognita* infection (Mi), *S. exigua* feeding (Se) and their interaction (Mi*Se) as the explanatory variables.

Plant traits	Source of variation	Df	Sum Sq	Mean Sq	Pseudo-F	R^2	P-value
JA, SA, ABA, *α*-tomatine	a). Invasion						
	Mi	1,16	0.04641	0.046407	1.52991	0.07675	0.235
	Se	1,16	0.06159	0.061591	2.03046	0.10186	0.135
	Mi*Se	1,16	0.01133	0.011327	0.37341	0.01873	0.812
	b) Galling						
	Mi	1,16	0.09426	0.094260	2.28677	0.11185	0.092
	Se	1,16	0.08055	0.080552	1.95422	0.09559	0.140
	Mi*Se	1,16	0.00838	0.008379	0.20328	0.00994	0.881
	c) Reproduction						
	Mi	1,16	0.89807	0.89807	36.717	0.63148	**0.001**
	Se	1,16	0.12972	0.12972	5.303	0.09121	**0.027**
	Mi*Se	1,16	0.00304	0.00304	0.124	0.00214	0.786

§ JA, jasmonic acid; SA, salicylic acid; ABA, abscisic acid; D.f., degree of freedom; Sum Sq; sum of squares; Mean Sq, mean of squares; Pseudo-F, F values by permutation; P, in boldface indicates statistical significance with $p < 0.005$; P- values based on 999 permutations (lowest P-value possible 0.001)

Table S3 Two-way factorial Analysis of Variance (ANOVA) results on phytohormone and *α*-tomatine for the herbivory effects of *Meloidogyne incognita* root-knot nematode (RKN) and the caterpillars of *Spodoptera exigua*. Root samples were collected from tomato plants infected with *M. incognita* at different life cycle stages. At the same time, root materials were collected from control and *S. exigua* infested plants. The impact of single and interactive effects of *M. incognita* and *S. exigua* feeding on jasmonic acid, salicylic acid, and abscisic acid were analyzed using linear models with *M. incognita* infection (Mi), *S. exigua* feeding (Se) and their interaction (Mi*Se) as the explanatory variables.

Hormone	Source of variation	Invasion			Galling			Reproduction		
		Df	F	P	Df	F	P	Df	F	P
JA	Mi	1,16	2.4379	0.1380	1,16	7.2298	**0.0161**	1,16	9.8473	**0.0064**
	Se	1,16	0.8640	0.3664	1,16	0.0101	0.9211	1,16	7.7380	**0.0133**
	Mi*Se	1,16	0.0843	0.7753	1,16	0.0208	0.8871	1,16	3.2416	0.0907
SA	Mi	1,16	0.0032	0.9557	1,16	0.3885	0.5419	1,16	24.3945	**0.0001**
	Se	1,16	1.0879	0.3125	1,16	2.1073	0.1659	1,16	2.3594	0.1441
	Mi*Se	1,16	0.2350	0.6344	1,16	1.5067	0.2374	1,16	0.7271	0.4064
ABA	Mi	1,16	1.2494	0.2802	1,16	1.3632	0.2601	1,16	64.8805	**5.07e-07**
	Se	1,16	1.0846	0.3132	1,16	2.3191	0.1473	1,16	1.4077	0.2528
	Mi*Se	1,16	1.1625	0.2969	1,16	0.0942	0.7629	1,16	0.3680	0.5526
α-tomatine	Mi	1,16	0.4026	0.5347	1,16	0.6014	0.4493	1,16	44.5357	**5.35e-06**
	Se	1,16	2.4654	0.1359	1,16	1.0287	0.3256	1,16	5.3249	**0.0347**
	Mi*Se	1,16	2.1021	0.1664	1,16	0.0058	0.9405	1,16	0.7079	0.4125

[§] JA, jasmonic acid; SA, salicylic acid; ABA, abscisic acid; dpi, day post nematode inoculation; Df, degree of freedom; F, F test value; P, P-value, in boldface indicate significant different $p \leq 0.05$

Table S4 Permutational Multiple Analysis of Variance (PERMANOVA) results based on Gower dissimilarities on gene expression data for herbivory effects of *Meloidogyne incognita* root-knot nematode (RKN) and the caterpillars of *Spodoptera exigua*. Root samples were collected from tomato plants infected with *M. incognita* at different life cycle stages. At the same time, root materials were collected from control and *S. exigua* infested plants. The single and interactive effects of *M. incognita* and *S. exigua* feeding on *Lipoxygenase D (LoxD); Leucine aminopeptidase A (LapA); Le4, abscisic acid-responsive Le4; and Basic-β-1-glucanase (GluB), jasmonate-responsive Ethylene Response Factor 4 (JRE4) and glycoalkaloids metabolism 1 (GAME1)* were determined via a MANOVA model with *M. incognita* infection (Mi), *S. exigua* feeding (Se) and their interaction (Mi*Se) as the explanatory variables

Gene	Source of variation	Df	Sum Sq	Mean Sq	Pseudo-F	R^2	P-value
LoxD, LapA, Le4, GluB, JRE4, GAME1	a). Invasion						
	Mi	1,16	0.02817	0.028169	0.80237	0.04007	0.556
	Se	1,16	0.05063	0.050630	1.44218	0.07202	0.222
	Mi*Se	1,16	0.06247	0.062470	1.77942	0.08886	0.121
	b). Galling						
	Mi	1,16	0.25410	0.254097	6.7827	0.25979	**0.001**
	Se	1,16	0.06018	0.060178	1.6063	0.06153	0.211
	Mi*Se	1,16	0.06440	0.064401	1.7191	0.06584	0.178
	c). Reproduction						
	Mi	1,16	0.30856	0.308560	11.0240	0.28658	**0.001**
	Se	1,16	0.27886	0.278864	9.9630	0.25900	**0.001**
	Mi*Se	1,16	0.04145	0.041447	1.4808	0.03849	0.225

§ *LoxD, Lipoxygenase D; LapA, Leucine aminopeptidase A; Le4, abscisic acid-responsive Le4; GluB, Basic-β-1-glucanase; JRE4, jasmonate-responsive Ethylene Response Factor 4; GAME1, glycoalkaloids metabolism 1*; D.f., degree of freedom; Sum Sq; sum of squares; Mean Sq, mean of squares; Pseudo-F, F values by permutation; P, in boldface indicates statistical significance with $p < 0.005$; P- values based on 999 permutations (lowest P-value possible 0.001)

Table S5 Two-way factorial Analysis of Variance (ANOVA) results on gene expression for the herbivory effect of *Meloidogyne incognita* root-knot nematode (RKN) and the caterpillars *of Spodoptera exigua.* Root samples were collected from tomato plants infected with *M. incognita* at different life cycle stages. At the same time, root materials were collected from control and *S. exigua* infested plants. The impact of single and interactive effects of *M. incognita* and *S. exigua* feeding on *Lipoxygenase D (LoxD); Leucine aminopeptidase A (LapA*); *Le4*, *abscisic acid-responsive Le4*; and *Basic-β-1-glucanase (GluB), jasmonate-responsive Ethylene Response Factor 4 (JRE4) and glycoalkaloids metabolism 1 (GAME1)* were analyzed using linear model with *M. incognita* infection (Mi), *S. exigua* feeding (Se) and their interaction (Mi*Se) as the explanatory variables.

Gene	Source of variation	Invasion			Galling			Reproduction		
		Df	F	P	Df	F	P	Df	F	P
LoxD	Mi	1,16	0.4725	0.5017	1,16	16.5670	**0.0009**	1,16	1.2412	0.2817
	Se	1,16	2.7393	0.1174	1,16	2.4657	0.1359	1,16	8.3360	**0.0107**
	Mi*Se	1,16	0.2754	0.6069	1,16	0.6747	0.4235	1,16	1.8034	0.1980
LapA	Mi	1,16	2.4068	0.1404	1,16	0.0001	0.9933	1,16	0.0775	0.7843
	Se	1,16	1.4937	0.2393	1,16	4.585	**0.0477**	1,16	14.6837	**0.0015**
	Mi*Se	1,16	5.5807	**0.0312**	1,16	2.8774	0.1092	1,16	2.3975	0.1411
Le4	Mi	1,16	2.7043	0.1196	1,16	2.3600	0.1440	1,16	3.4082	0.0835
	Se	1,16	0.0823	0.7778	1,16	0.0100	0.9217	1,16	0.0159	0.9012
	Mi*Se	1,16	0.0584	0.8121	1,16	2.8430	0.1112	1,16	2.4099	0.1401
GluB	Mi	1,16	1.2921	0.2724	1,16	0.9654	0.3404	1,16	1.5753	0.2275
	Se	1,16	0.7648	0.3948	1,16	0.1532	0.7006	1,16	13.8329	**0.0019**
	Mi*Se	1,16	0.8576	0.3682	1,16	0.6618	0.4279	1,16	2.1365	0.1632
JRE4	Mi	1,16	0.2127	0.6509	1,16	15.8712	**0.0011**	1,16	30.2022	**4.89e-05**
	Se	1,16	0.0139	0.9078	1,16	1.3143	0.2685	1,16	0.0177	0.8957
	Mi*Se	1,16	4.6575	**0.0465**	1,16	2.5703	0.1284	1,16	4.7645	**0.0443**
GAME1	Mi	1,16	0.3679	0.5527	1,16	6.4438	**0.0219**	1,16	68.9237	**3.42e-07**
	Se	1,16	4.2435	0.0561	1,16	2.7830	0.1147	1,16	15.1963	**0.0013**
	Mi*Se	1,16	0.2399	0.6309	1,16	0.6425	0.4345	1,16	0.0708	0.7936

§ *LoxD, Lipoxygenase D; LapA, Leucine aminopeptidase A; Le4, abscisic acid-responsive Le4; GluB, Basic-β-1-glucanase; JRE4, jasmonate-responsive Ethylene Response Factor 4; GAME1, glycoalkaloids metabolism 1*; dpi, days post nematode inoculation; Df, degree of freedom; F, F test value; P, P-value, in boldface indicate significant different $p \leq 0.05$

References

Abdelkareem A, Thagun C, Nakayasu M, Mizutani M, Hashimoto T, Tsubasa Shoji. **2017**. Jasmonate-induced biosynthesis of steroidal glycoalkaloids depends on COI1 proteins in tomato. *Biochemical and Biophysical Research Communications* **489**: 206–210.

Martínez-Medina A, Fernández I, Sánchez-Guzmán MJ, Jung SC, Pascual JA, Pozo MJ. **2013**. Deciphering the hormonal signalling network behind the systemic resistance induced by *Trichoderma harzianum* in tomato. *Frontiers in Plant Science* **4**: 1–12.

Uppalapati SR, Ayoubi P, Weng H, *et al.* 2005. The phytotoxin coronatine and methyl jasmonate impact multiple phytohormone pathways in tomato. *The Plant Journal* **42**: 201–217.

Yan L, Zhai Q, Wei J, *et al.* 2013. Role of tomato *lipoxygenase D* in wound-induced jasmonate biosynthesis and plant immunity to insect herbivores. *PLoS genetics* **9**: 1–16.
